# Direct conversion of fibroblasts to osteoblasts as a novel strategy for bone regeneration in elderly individuals

**DOI:** 10.1038/s12276-019-0251-1

**Published:** 2019-05-09

**Authors:** Yujung Chang, Byounggook Cho, Siyoung Kim, Jongpil Kim

**Affiliations:** 1 Department of Biomedical Engineering, Dongguk University, Pildong-ro 1-gil 30, Jung-gu, Seoul, 04620 Republic of Korea; 2 Department of Chemistry, Dongguk University, 30, Pildong-ro 1-gil 30, Jung-gu, Seoul, 04620 Republic of Korea

**Keywords:** Reprogramming, Regeneration

## Abstract

Mortality caused by age-related bone fractures or osteoporosis is steadily increasing worldwide as the population ages. The pace of the development of bone regeneration engineering to treat bone fractures has consequently increased in recent years. A range of techniques for bone regeneration, such as immunotherapy, allografts, and hydrogel therapy, have been devised. Cell-based therapies using bone marrow-derived mesenchymal stem cells and induced pluripotent stem cells derived from somatic cells are considered to be suitable approaches for bone repair. However, these cell-based therapies suffer from a number of limitations in terms of efficiency and safety. Somatic cells can also be directly differentiated into osteoblasts by several transcription factors. As osteoblasts play a central role in the process of bone formation, the direct reprogramming of fibroblasts into osteoblasts may hence be a new way to treat bone fractures in elderly individuals. Here, we review recent developments regarding the therapeutic potential of the direct reprogramming of cells for bone regeneration.

## Fractures: reprogramming cells to reduce risk in the elderly

Reprogramming cells that produce connective tissue to form bone instead could help prevent fractures in the elderly. Bones weaken with age, and fractures are a significant health risk in ageing populations. Most current bone regeneration treatments use stem cells, which can differentiate into any type of cell and have infinite capacity to divide; however, they are difficult to source and can lead to tumor formation. Jongpil Kim at Dongguk University in South Korea and coworkers have reviewed a new method that uses genetic signals to transform connective tissue-forming cells into bone-producing cells. The reprogrammed cells have been shown to generate new bone at the desired site, and because they have already lost their capacity for infinite division, tumor formation risk is greatly reduced. This method shows promise to expand treatment options for fractures and osteoporosis.

## Introduction

Most elderly people develop osteoporosis with age. Osteoporosis results in bone loss and an increased risk of fractures that can lead to death^1,2^. Several techniques for promoting bone regeneration and repair have been developed to reduce bone fracture-related mortality in elderly individuals. These techniques include allografts^3^, gene therapy^4^, and cell-based therapy, all of which have undergone testing in patients^5,6^. Recent studies have highlighted that somatic cells can readily be converted into specific cell types without the involvement of a pluripotent state^7,8^. This reprogramming can directly induce the formation of the intended cell type from somatic cells, whereas it can also be used to safely generate substantial amounts of the desired cell type^9^. Thus, there have been a number of attempts in recent years to treat several diseases using direct cell reprogramming techniques^10,11^.

Osteoblasts play an important role in bone regeneration, as these cells generate factors, such as osteopontin, that are involved in the induction of bone development. The successful direct conversion of fibroblasts into osteoblasts has been reported using defined transcription factors, such as Osterix, runt-related transcription factor 2 (Runx2), octamer-binding transcription factor 3/4 (Oct4), and L-myc^12^. These findings indicated that osteoblasts can readily be obtained without the induction of a pluripotent state. In addition, these osteoblasts were shown to promote new bone formation at the fracture site. In this review, we highlight the advantages of direct reprogramming into osteoblasts. Furthermore, we propose that the direct conversion of fibroblasts into osteoblasts to improve bone regeneration in elderly individuals warrants clinical application.

## The importance of bone homeostasis: osteoblast–osteoclast relationship

Bone is considered a structurally and functionally complex tissue^13^. The mechanical properties of the >200 bones of the human skeletal system are largely influenced by intrinsic and extrinsic conditions of the environment^13,14^. Bone is embedded in the extracellular matrix and comprises a vast network of canaliculi filled with a special fluid^15,16^. Bone homeostasis consists of two processes: bone formation by osteoblasts and bone resorption by osteoclasts. Osteoblasts, which are generated from mesenchymal stem cells (MSCs), play a crucial role in the maintenance and regeneration of bone mass, the determination of bone quality, and the functioning of the skeletal system^17–20^. The main role of osteoblasts is to synthesize and secrete a range of proteins involved in bone formation (e.g., extracellular matrix proteins, cytokines, collagen, and growth factors) and to convert extracellular matrix into bone by mineralization^21^. Before the organic matrix of bone is formed, this component is called the osteoid. In general, mineralization of the osteoid depends on Ca^2+^ in the plasma^22,23^. The function and activity of osteoblasts are influenced by a variety of factors, including transcriptional and epigenetic mechanisms^24^, cell–cell interactions^25^, cell–matrix interactions^26^, and inflammatory processes^27^. Thus, there is an abundance of evidence indicating that osteoblasts are critical for bone formation. In contrast, osteoclasts are responsible for bone resorption, which results in the breakdown of bone tissue^28^. Osteoclasts are found in small depressions on the surface of the bone, called Howship lacunae^29,30^. This deposition is triggered by an erosion of the bone by osteoclast enzymes. Osteoclasts produce a variety of enzymes that dissolve the bone matrix and calcium of the bone. Mineralized bone is broken down and the collagen fibrils are engulfed by osteoclasts^31,32^. Thus, bone is an energetic tissue that is consistently being broken down and remodeled by the activities of osteoblasts and osteoclasts.

## Association of osteoporosis and bone fractures with aging

**Fig. 1:**
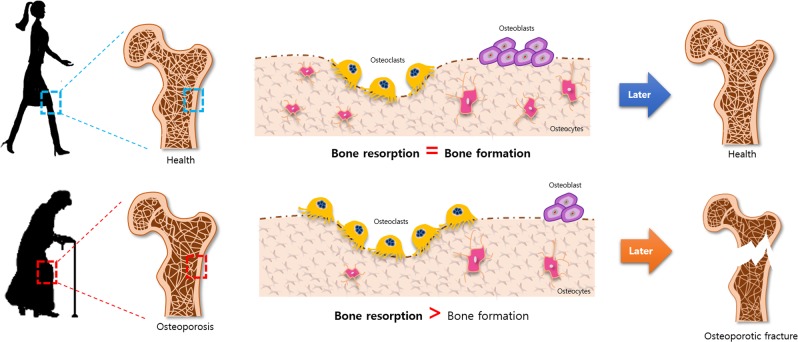
Bone remodeling. Bone formation and resorption are mainly regulated by the interplay between osteoblast differentiation and osteoclast activation. With increasing age, the balance shifts to more bone resorption than bone formation

The incidence of aging-associated bone loss has increased worldwide^2^. Recent surveys have shown that ~ 20% of people over the age of 50 will experience an osteoporotic fracture at one stage or another. Osteoporotic hip fractures account for ~ 30% of all bone fractures in elderly individuals^33^. Bone fractures are one of the most-devastating consequences of osteoporosis, as they often lead to an increase in morbidity and mortality^1^. Most elderly people will experience a fracture, and studies in the USA have indicated that the number of such fractures has been increasing each year. As a result, aging-related fractures have become one of the main contributors to the increase in health-care costs in recent years^34^. Osteoporosis in elderly individuals is characterized by inadequate bone mass owing to changes in sex hormones, inflammation, and various metabolic complications^35,36^. Aging results in intrinsic senescence-related mechanisms that trigger oxidative stress and changes in bone homeostasis that affect the generation and apoptosis of osteoblasts^37^. These factors contribute to impaired osteoblastogenesis. With advancing age, the structure and elasticity of the bone matrix change^38^ and bone mass decreases owing to an imbalance caused by reduced bone formation and increased resorption^39^. Moreover, bone formation and resorption are mainly regulated by the interplay between osteoblast differentiation and osteoclast activation^40,41^. With increasing age, the balance shifts to more bone resorption than bone formation (Fig. 1). Therefore, finding ways to increase bone repair by the differentiation of progenitor cells into osteoblasts, promoting endochondral ossification, and increasing cartilage formation, are among the main objectives of current bone research.

## Bone tissue engineering to treat bone fractures

A number of different approaches have been taken to promote bone repair, including the local application of osteogenic bone marrow-derived cells^42^, transplantations using autografts and allografts^3,41–43^, gene therapies^4^, and the use of biomaterials^44,45^ (Table 1). The standard method for bone repair is the implantation of autogenous bone, which is transferred from one site to another in the same patient^46^. However, this method is subject to considerable limitations, such as graft resorption, donor site morbidity, limited availability, and the operative time for harvesting grafts^47^. Alternatives to autografts, such as allografts and xenografts to treat bone defects, have become available^46^. However, both of these alternatives are rarely used owing to their high costs, possibility of disease transmission, lack of osteoinduction, and immunogenesis^47,48^. Immune engineering has been used to alter the immune system to boost bone repair and regeneration after bone fracture or injury^49^. A bone fracture attracts an abundance of inflammatory cytokines and growth factors in addition to promoting the differentiation of MSCs^49,50^. These factors play a critical role in bone remodeling as a result of the formation and resorption of osteoblasts and osteoclasts, respectively. A healing bone fracture can, however, be impaired by aberrant inflammation and autoimmune disorders that suppress osteogenesis^49^. Hydrogels, which comprise a group of polymeric materials, have been reported to promote bone tissue regeneration as a result of their ability to mimic the extracellular matrix topography and deliver bioactive agents^51^. Nevertheless, the use of hydrogels suffers from difficulty in controlling the release of encapsulated bioactive molecules, and these compounds have also resulted in toxic reactions due to the use of inappropriate polymers^52^. Bone marrow-derived MSCs (BM-MSCs) have become one of the mainstays of current cell-based therapies^53,54^. For example, experimental findings and clinical cases indicate that BM-MSCs, owing to their osteogenic potential, can be transplanted to repair bone defects^55,56^. Moreover, the treatment of BM-MSCs with epigenetic modulators, such as agents that decrease genomic DNA methylation, has been reported to promote osteogenic differentiation and enhance osteogenic gene expression^57,58^. However, the use of epigenetic modulators in clinical application for bone regeneration is restricted in elderly people due to the limited yield and expandability of BM-MSCs, and the ability of these cells to differentiate into osteoblasts. At present, induced pluripotent stem cells (iPSCs) have been recognized for their ability to differentiate into a range of different cell types that can be used in bone tissue engineering^6,59–63^. Human iPSCs, for instance, have been differentiated toward bone fate, expressing osteogenic markers, such as RNUX2, OSX, OCN, BSP, and Col1a1^64^. Furthermore, the in vivo transplantation of iPSC-derived osteoblasts has been reported to lead to mineralization and the formation of bone matrix^64^. Thus, stem cell-based therapies have the potential to be applied in the treatment of bone defects. However, stem cell-based therapies suffer from several limitations, including time-consuming procedures, ethical and safety concerns related to their transplantation into humans, immunological rejection responses, and possibility of teratoma formation^65–67^.

**Table 1:** The table shows the pro and cons of osteogenic cells derived from different sources

The method of bone repair		Pros	Cons	Ref.
Transplantation	Autograft	No tissue rejection	Donor site morbidity	
		Lower infection rate	Limited availability	
		Lower cost	Graft resorption	^46^
	Allograft	No zoonosis	Immunological rejection	^47^
			High expense	^48^
			High morbidity	
	Xenograft	Mass production	Disease transmission	
Biomaterials		Biodegradable materials	Toxic reaction	^51^
		Biocompatibility	Difficulty in handling	^52^
Bone marrow- derived mesenchymal stem cells (BM-MSCs)		Self-renewal differentiation ability of various cell types	Limited source of tissue	^55^
				^56^
				^57^
				^58^
Induced Pluripotent Stem cell (iPSCs)		High proliferation	Require many steps to manufacture	^64^
		Low immune rejection	Risk of teratoma formation	^65, 66^
Direct conversion		Low risk of mutation	Lack of targeted cell types	^77^
		Generation of specific cell type low immune rejection	Low efficiency aged reprogrammed cells	^78^

## Direct reprogramming

The conversion of somatic cells into specific cell types without passing through an intermediate stage by the introduction of combinations of lineage-specific factors is called direct reprogramming. Direct reprogramming was first introduced by Davis et al.^68^, who demonstrated that fibroblasts can be converted into myoblasts by the ectopic expression of the muscle-specific transcription factor *MyoD.* Recent studies have reported that specific transcription factors can induce somatic cells to form several cell types, including cardiomyocytes^7,8^, neurons^69^, hematopoietic progenitor cells^70^, and pancreatic beta cells^71^, without a transient pluripotent stage. For instance, three transcription factors, Ascl1, Brn2, and Mty1l, can efficiently induce the formation of functional neurons from fibroblasts, resulting in the expression of neuronal proteins and the generation of action potentials^72^. In addition, three transcription factors, namely, Gata4, Hnf1a, and Foxa3, have been reported to induce the formation of functional hepatocyte-like cells (iHep) from mouse fibroblasts. The resulting iHep cells express hepatic genes and show an epithelial morphology^73^.

Several studies have shown that a combination of specific factors can reprogram specific functional cell types in vivo^71,74^. For example, studies have shown that pancreatic exocrine cells in adult mice can be converted into β-cells by the injection of three factors, such as Ngn3, Pdx1, and Mafa, thereby suggesting a potential application of in vivo reprogramming for type I diabetes^71^. In another study, cardiac fibroblasts could be induced to form cardiomyocytes by the delivery of cardiac transcription factors, such as Gata4, Mef2c, and Tbx5, in the mouse heart after coronary ligation, thus demonstrating novel strategies for the treatment of cardiac disease^74^.

The direct reprogramming technique has several potential advantages over the use of iPSCs. A previous study found that iPSCs have numerous genomic aberrations and that these cells undergo changes in gene copy numbers during their passage and differentiation^75^. The increase in gene copy numbers with pluripotency and cell proliferation enhances the risk of oncogenesis^75,76^. There is, however, less risk of such mutations with direct reprogramming because this process can take place in the absence of cell proliferation^77,78^. In addition, the proliferation of iPSCs in the uncontrolled state is similar to that of cancerous cells^79^. Thus, by avoiding full pluripotency, the conversion of cell fate by direct reprogramming means that this process has a lower risk of tumor formation^80^. For this reason, direct conversion to generate patient-specific cells has ample potential to lead the development of clinically applicable cell therapies^81,82^. Thus, direct reprogramming is more technologically advanced for clinical applications^83,84^. Moreover, in vitro and in vivo direct conversion technologies may be useful for regenerative therapy because the direct conversion into specific mature cell types is more efficient at producing functional mature cells without having to involve a pluripotent stage^85,86^.

## Direct reprogramming into osteoblasts

Generally, a combination of transcription factors has been used to reprogram fibroblasts into osteoblasts. In 2015, Yamamoto et al.^12^ first reported that human fibroblasts can be directly reprogrammed into osteoblasts using transcription factors, such as Runx2, Osterix, and Oct3/4, L-Myc (RXOL). The authors selected these factors as regulators of osteoblast development that have the ability to determine cell fate into osteoblasts. After the transduction of RXOL, the authors detected induced osteoblast-like cells (iOBs) with an osteogenic morphology, the production of bone matrix, and the expression of osteoblast-related genes. Despite the heterogeneity of the iOB population, the overall gene expression profile of RXOL-induced osteoblasts was similar to that of osteoblasts. These researchers demonstrated that ROXL-reprogrammed cells did not pass through an intermediate pluripotent cell type, as immunostaining with anti-Nanog for 15 days indicated that Nanog was not expressed. The iOBs were transplanted at a site with a bone defect in an immunodeficient mouse model, resulting in callus formation, followed by ossification at the iOB-transplanted site. Thus, the direct conversion of somatic cells into osteoblasts using Runx2, Oct4, Osterix, and L-myc represents a feasible cell-based therapy under bone resorption conditions^12^. In that same year, this research group also suggested that the transduction of Oct9 and N-myc could convert human fibroblasts into osteoblast-like cells, thereby inducing an osteoblast-like phenotype and the expression of Runx2 and osteocalcin^87^.

**Fig. 2:**
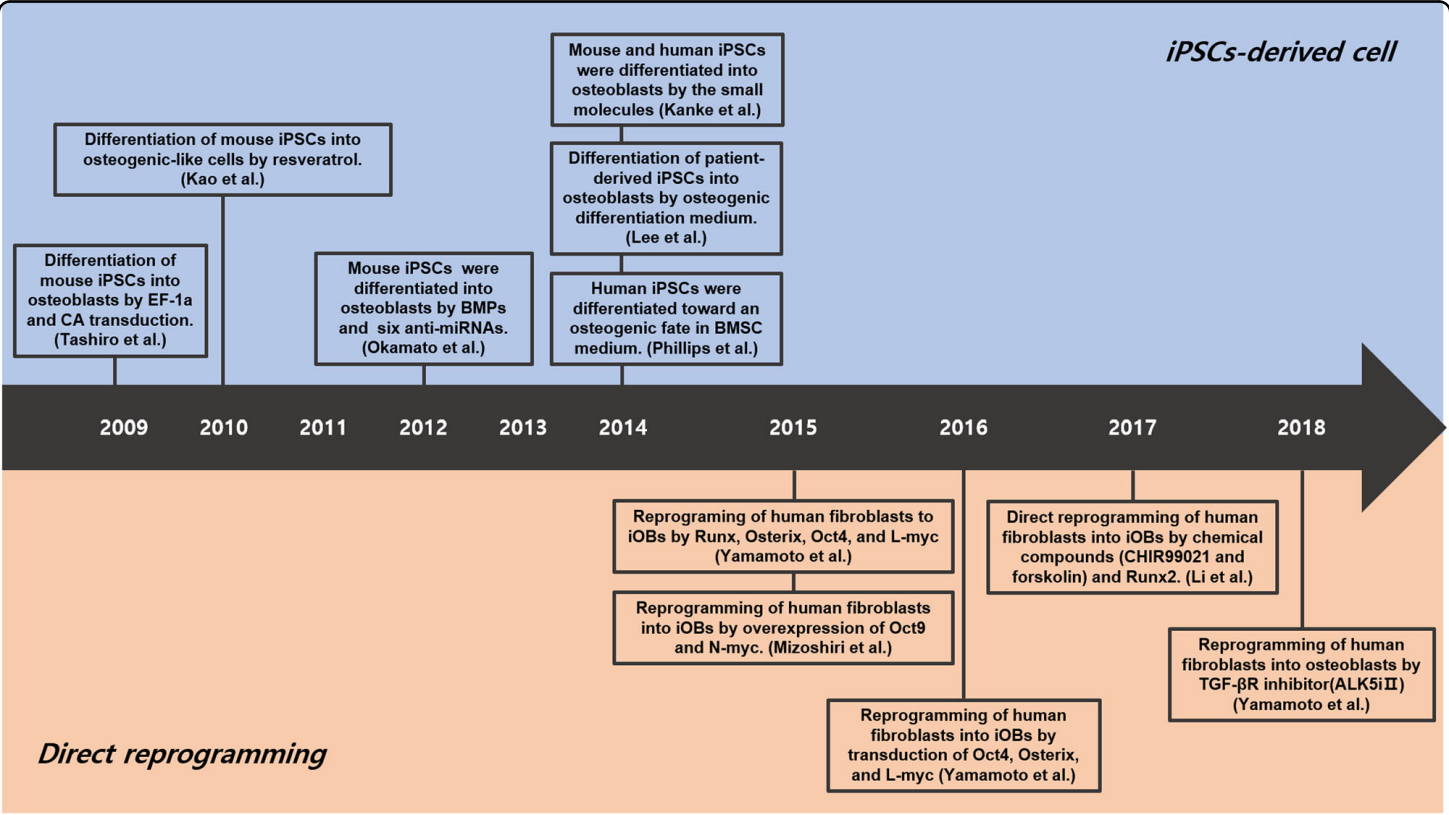
Timeline for the development of osteogenic cells. The upper panel shows the development of iPSC-derived osteogenic cells, whereas the lower panel shows the development of direct conversion-derived osteogenic cells

In a subsequent publication, Yamamoto et al.^88^ also indicated that human fibroblasts can be directly converted into osteoblasts using plasmid vectors encoding Osterix, L-myc, and Oct4. The effectiveness of this procedure was demonstrated by bone matrix production and osteoblast-specific gene expression in culture^88^. In addition, as an alternative to transcription factor-mediated reprogramming, Yamamoto et al.^89^ also showed that osteogenic transcription factors can be substituted by specific chemical compounds. These authors identified 12 chemical compounds that contribute to the maintenance of pluripotency and the development of osteoblast-like cells, suggesting that the direct conversion of fibroblasts into osteoblasts may be possible. Among these compounds, the TGF-β inhibitor ALK5iII induced a significant degree of osteogenic reprogramming, as the resulting iOBs exhibit a degree of calcium deposition and high ALP activity. Intriguingly, the transplantation of chemically induced osteoblasts at a site with an artificial bone defect resulted in massive callus formation and ossification in immunodeficient mice. Thus, the direct conversion of the cells surrounding a fracture may be a viable approach for promoting bone formation by osteoblasts following an injury (Fig. 2). Although the effectiveness of directly reprogrammed osteoblasts has not been fully demonstrated to date in clinical studies, this technique has the potential to allow for the efficient and safe treatment of bone fractures in elderly patients.

## Biosafety issues for bone therapy using direct cell reprogramming

Although direct reprogramming holds great promise for regenerative medicine, ensuring the safety of directly converted cells has faced many obstacles in clinical settings. The introduction of viruses in humans is generally not considered a viable option. As transcription factors integrate into the genome, cells with the insertion of such transgenes into their DNA may undergo neoplastic transformation or lead to inflammatory disease and epigenetic abnormalities^90–93^. For example, virus-mediated reprogrammed cells have been shown to undergo the genomic integration of reprogramming factors^94^. Therefore, an alternative method is needed before entities, such as viral-induced cells, are used in clinical applications. The methods based on entities other than transcription factors, such as modified RNA, growth factors, and small molecules, need to be considered.

Moreover, a fundamental problem with the direct reprogramming of somatic cells into specific cells for use in patients is telomere shortening, which results in genetic stability. A previous study reported that directly reprogrammed cells have preserved features, such as DNA damage, the proportion of heterochromatin, nuclear organization, and telomere lengths^95^. In this study, induced cells obtained by the direct reprogramming of old cells retained aged phenotypes, including an aberrant nuclear morphology and a high level of DNA damage. In addition, a lack of the required cell type and limited proliferation could also be major concerns with the application of direct lineage reprogramming for bone regeneration. Thus, a strategy for controlling telomere shortening and genetic stability, as well as the expansion of a large number of cells, is important in the direct reprogramming of fibroblasts into osteoblasts to treat elderly patients with cell-based therapies. Moreover, it has yet to be demonstrated that osteoblasts generated in this manner are functionally similar to bona fide osteoblasts. A previous study found that the direct reprogramming of osteoblasts from fibroblasts using transcription factors yielded only a small number of reprogrammed cells and resulted in the low expression of endogenous osteogenic genes in iOBs owing to incompletely functional direct conversion^12^. Moreover, for the in vivo reprogramming of osteoblasts as a clinical application, efficient in vivo reprogramming methods in an in vivo environment should be considered. In addition, a controllable expression system including the reprogramming factors in an in vivo environment is essential for optimal in vivo reprogramming induction. Thus, future studies are needed to fully characterize the nature and function of osteoblasts generated by direct reprogramming before clinical applications can take place.

## Conclusions

As the population ages, bone fractures caused by age-related osteoporosis have become a very significant problem, and there is a pressing need for new treatment modalities. The possibility of altering the function and activity of osteoblasts to treat bone fractures has been investigated extensively. Direct reprogramming of somatic cells into osteoblasts represents a potential approach for the safe and efficient treatment of bone fractures in elderly individuals. In this review, we propose several clinical applications of a direct conversion method for generating osteoblasts in patients. Future work is needed to determine the best way to directly reprogram somatic cells into osteoblasts for optimal clinical use. In addition, the optimal cellular microenvironment must be considered for direct conversion that promotes osteoblast survival and bone formation in patients. However, the transplantation of directly reprogrammed osteoblasts or in vivo direct osteogenic reprogramming has ample potential to become an alternative to autologous bone grafts for treating bone fractures. Indeed, with further advances in this area, the direct reprogramming of somatic cells into osteoblasts may become the treatment of choice for bone fractures in elderly individuals.
